# Comparison of Health Status and Health Care Services Utilization between Migrants and Natives of the Same Ethnic Origin—The Case of Hong Kong

**DOI:** 10.3390/ijerph10020606

**Published:** 2013-02-04

**Authors:** Su Liu, Catherine X. J. Hu, Selene Mak

**Affiliations:** JC School of Public Health and Primary Care, Chinese University of Hong Kong, Prince of Wales Hospital, Shatin, N.T., Hong Kong; E-Mails: sliu@cuhk.edu.hk (S.L.); catherinehu@cuhk.edu.hk (C.X.J.H.)

**Keywords:** migrants, primary care, utilization, disparity, social determinant of health, Hong Kong, China

## Abstract

Based on the 2009 Thematic Household Survey in Hong Kong, this study compared health status and utilization of health care services in Hong Kong between migrants from Mainland China and natives. Overall, Mainland migrants reported lower socioeconomic conditions, worse health status, and less health care services utilization than the natives. After controlling for socio-demographic factors, we found that the migrants were 1.2 times more likely to report fair or poor health and 0.78 times less likely to report having a usual source of care, compared with the natives. Mainland migrants also had fewer physician visits and relied more on the public sector. Within the migrant group, those who had language advantage had more visits, and the recent arrivals who stayed in Hong Kong for three years or less had fewer visits and were far less likely to have a usual source of care. The findings underscore migration as an important social determinant of health in Hong Kong. A combination of targeted social and health policies is needed to help Mainland migrants better integrate into society and to improve their access to care. Programs should be tailored to address varying needs from different subgroups among migrants.

## 1. Introduction

Migrant health has become a growing field of research in recent years [1,2,3,4,5]. It is well-recognized that migration is an important social determinant of health [6,7,8]. From a broader perspective, research on migrant health has widespread implications that extend beyond effects applicable only to migrants. The changes that a country experiences as a result of migration affect the health and well-being of migrants as well as the native population. 

Much of the research on migrant health has focused on people of different race and ethnicity migrating to a foreign county. A growing body of literature has reported newly arrived migrants in better health than natives, but this migrant health advantage eventually deteriorates over time [9]. This phenomenon, known as the “healthy migrant hypothesis”, has been documented extensively using data on migrants to the United States [10], Canada [11], Australia [12], and Europe [13,14].

There are, however, fewer studies discussing the “healthy migrant hypothesis” and health care utilization in the context of internal migration. Internal migration can be defined differently under various settings. Existing studies have generally discussed the rural-to-urban migration with the backdrop of fast economic development in countries such as China and India [15,16,17,18]. Researchers have, in recent years, also included place of origin, such as countryside, township, or city, in discussion of internal migration [15]. Despite different focuses and context, internal migration has been commonly defined within the same country’s border. 

The process in which people from Mainland China migrate to Hong Kong Special Administration Region (HKSAR) requires full immigration protocol under HKSAR’s “one country, two system” government. Though moving within the same country, these migrants are moving to a place with a very different legal, political, and health care system. In fact, the process mimics an international migration. However, unlike most international migrants, who are by definition genetically and culturally different than the native population, migrants from Mainland China in most cases are of the same race/ethnicity origin and share similar culture as the natives born in Hong Kong. How will this affect their health and health care utilization? Will the same evidence from the literature on international and internal migrants’ health (*e.g*., the healthy migrant hypothesis) be found here? We will address these questions focusing on migrants of same ethnic origin moving across a significant administrative boundary through the unique case of Hong Kong. 

### 1.1. Background: Immigration and Health Care System in Hong Kong

Hong Kong, with land area of 1,104 square miles and about seven million residents, was a British colony until 1997. In the 1950s, despite the Hong Kong government’s effort to stem the wave of illegal immigrants from Mainland China by negotiating a quota system that would allow a daily number of Mainland residents to enter Hong Kong, it did not stop illegal immigrants fleeing economic and political hardship during the Communist Revolution from entering the territory. In the late 1970s and early 1980s, around half a million Mainland residents, mostly illegal, entered Hong Kong as a result of China relaxing its border controls. This group of migrants was eventually granted legal residence by the Hong Kong government [19]. 

As these new immigrants became more successful, they returned to Mainland to find wives, which would explain why many of the one-way permits (OWP) issued in late 1980s and the 1990s were for family reunification purposes. Government statistics show that most OWP were issued to “mostly dependent women with few marketable skills and burdened by young children” who had married Hong Kong residents [19]. Some of these immigrants have had trouble integrating into Hong Kong society. Language barriers and lack of family support have been cited as reasons for the difficulty. Over time, particularly after the return to China in 1997, the types of immigrants entering Hong Kong have diversified and included more individuals with higher education and better socioeconomic status, through programs such as the Admission Scheme for Mainland Talents and Professionals, the Quality Migrant Admission Scheme, or the Capital Investment Entrant Scheme. 

The current “one country, two system” governing structure created the HKSAR, which grants authority to the local government to make immigration-, education-, and health care-related policies. Hong Kong has a two-pillared health care system comprised of a government-financed public health care system and a loosely-regulated private health care system. The public system, providing mainly inpatient care and some outpatient care, covers anyone with a Hong Kong identification card (including legal immigrants with established residential status) or children under the age of 11 with resident status. The majority of outpatient care services, however, are delivered by private clinics. As a result, provision of care is fragmented and is focused on treatment instead of prevention. The concept of primary care and having a regular doctor has neither been widely appreciated by the public nor practiced among providers. In fact, any registered doctors, dentists, and practising Chinese medicine practitioners in Hong Kong with *self-declared* commitment to the provision of directly accessible, comprehensive, continuing, co-ordinated, and person-centred primary care services are currently eligible for enrolment in the Government’s Primary Care Directory [20]. People seek ambulatory care from all types of public or private doctors practicing western allopathic or traditional Chinese medicine that they may have access to. Realizing effective primary care can lead to better health outcomes, lower costs, and greater equity in health, the Hong Kong government established a Primary Care Office under the Department of Health in September 2010 to lead its own primary care development [20,21,22].

### 1.2. Objectives

According to the Hong Kong Census and Statistics Department (HK C&SD), there are 2,793,450 foreign-born people now living in Hong Kong, constituting about 40% of the total population [23]. The number of foreign-born will continue to rise as HKSAR continues to attract talent and capital to the territory through several General Employment Policy (GEP) initiatives. For instance, in 2011, the Admission Scheme for Mainland Talents and Professionals admitted about 50,000 people and the Quality Migrant Admission Scheme admitted 2,000 people to Hong Kong [24]. Based on the mutual agreement between the Mainland China and Hong Kong, the quota for One-way Exit Permit is 150 per day, or 54,750 per year [25]. These demographic trends suggest that the health status and health care utilization patterns of Mainland migrants and their households will play an increasing role in shaping Hong Kong’s health policy.

Using data from 2009 Thematic Household Survey (THS), this paper looked at three research questions concerning migrants from Mainland China and Hong Kong natives:

(1) Does the “healthy migrant hypothesis” hold for new migrants and disappear over time, as found in the international migration literature?
(2) How does health care utilization differ between natives and migrants?
(3) Are there other facilitators or barriers explaining utilization difference (if any) among migrants, such as language ability?

Findings will not only help inform the ongoing population and health policy development, particularly the primary care reform in Hong Kong, but will also provide much-needed evidence in the global literature on migration of the same ethnic origin across a significant administrative border.

## 2. Methods

### 2.1. Data Source and Study Population

The Census and Statistics Department of HKSAR has conducted a series of territory-wide cross-sectional Thematic Household Survey to collect statistical data on different social topics periodically since 1999. This study was based on the round of THS conducted during November 2009 to February 2010, which collected information on health-related topics, including the health status and health care utilization of Hong Kong residents. In addition, respondents are asked to provide socioeconomic characteristics, including place of birth, length of stay in Hong Kong, age, sex, marital status, level of education, and income. The face-to-face survey included 10,028 households representing land-based non-institutional population of Hong Kong, with a response rate of 75% [26].We used age, place of birth, and length of stay in Hong Kong as inclusion criteria to obtain our study population—a total of 23,040 individuals who were aged 18 and above, either born in Hong Kong or Mainland China, and have been in Hong Kong for at least 90 days were included. Children, migrants from other parts of the world, and short-term visitors were excluded. 

### 2.2. Definition of Comparison Groups and Hypotheses

To answer the three research questions, we further divided the study population into comparison groups. The key comparison is between Hong Kong natives (n = 15,305, 66.4%) and Mainland China migrants (n = 7,735, 33.6%), which was defined based on responses to the question “Where were you born”. Migrants were further categorized by their length of stay (<4, 4–7, 8–10, 11–20, 21–30, 31–40, 41–50, 51–60, 61+) based on their answer to the question “For how many years have you been living in Hong Kong”. Those who have been in Hong Kong for three years or less will be referred to as “recent arrivals” hereafter (n = 293, 3.8%). Based on the existing research, they have most trouble integrating in the society, therefore, will be a key study subgroup and compared with those who have been living in Hong Kong longer [27,28]. Finally, using province of birth as proxy for the primary language spoken, we will compare migrants from the neighboring province, Guangdong (n = 6,102, 78.9%) who speak Cantonese—the main spoken language in Hong Kong, and migrants from other parts of Mainland China (n = 1,633, 21.1%) who speak Mandarin, to ascertain whether language poses a barrier to health service utilization.

Based on these comparison groups and the existing literature, we hypothesized that: (1) Recent arrivals will have better health, but such an advantage will disappear as they stay longer in Hong Kong; (2) Migrants will have lower service utilization than the natives; and (3) Migrants from Guangdong province will have higher utilization compared with other migrants. 

### 2.3. Outcome Measures

We used self-reported overall health condition as the primary outcome measure for health status. Respondents were asked “overall speaking, what do you think about your health condition” and presented with the following choices: excellent, very good, good, fair, or poor. We created a dichotomous variable by grouping fair and poor together. We also looked at whether respondents were current smokers, whether respondents were currently diagnosed with diabetes, hypertension, or coronary artery disease, through the question “have you ever been told by a Western medicine practitioner that you had the following chronic health conditions”. 

For health care utilization, our primary outcome measure was number of doctor visits. Respondents were asked “in the past 12 months, have you had a consultation with a doctor either inside or outside Hong Kong?” and if “yes”, “which of the following types of clinics have you visited? For every type, approximately how many times have you visited?” We added up all the visits, regardless of types of clinics, to come up with number of total visits. In addition, we zoomed in on western medicine to look at the split between the public and private sector through three measures: number of western medicine consultations occurred in the public clinics and private clinics, respectively, as well as the percent of such visits in the public sector. Considering the current practice in Hong Kong, we tried to interpret these results in a loosely defined “primary care” context. Furthermore, we also considered whether respondents had a usual source of care and looked at number of accident and emergency (A&E) visits, in order to better understand the more strictly defined “primary care” known as accessible first contact care that is comprehensive, continuing, coordinated, and person-centered [20,21]. 

### 2.4. Statistical Analysis

The above health status and utilization measures were summarized for each comparison group defined by migration status, birth province, or length of stay. Because the demographic profile of these comparison groups are likely to vary and drive the health-related outcomes, descriptive statistics of the primary outcome measures were presented per age-sex category. Group differences were evaluated using Chi-square test for categorical variables and Independent T-test or ANOVA for continuous variables. Unless otherwise noted, differences between comparison groups, as reported in the results section, are statistically significant at 5% level.

In addition to binary statistics, we also conducted multivariate analyses for selected outcome measures to examine if any difference existed in health status or utilization between Mainland migrants and Hong Kong natives, after (1) adjusting for age and sex, and (2) controlling for other socioeconomic characteristics (e.g., marital status, education, household income, employment status) and health-related factors (e.g., smoking). By including two additional 0/1 migration factors as covariates—one for someone from Guangdong province (a proxy for minimum language barrier) and the other identifying the recent arrivals—we could also investigate how language and length of stay might affect the outcomes. 

Logistic regression was conducted to look at the two binary outcomes, *i.e.*, self-reported having fair/poor health and having usual source of care. For total number of doctor visits, which was skewed to the right with a large variance, we chose to use negative binomial regression, since it is more appropriate for analyzing overdispersed count data [29]. Finally, we used a generalized linear model (GLM) with a logit link and the binomial family, to analyze the percent of visits to western medicine doctors that occurred in the public sector, taking into account its nature of proportion data with values that fall between zero and one [30]. In case we misspecified the distribution family in the GLM, robust standard errors were obtained. All regression models were estimated by STATA [31].

## 3. Results

We will first report the demographics and key outcome measures for the comparison groups, followed by presentation of the results from descriptive and multivariate analyses of the health status and health care utilization, respectively. 

### 3.1. Comparison of Demographics and Socioeconomic Conditions

Compared with Hong Kong natives, migrants from China were older, particularly those from Guangdong, a finding consistent with the earlier migration waves (Table 1). Migrants were also more likely to be female, and currently married, particularly the recent arrivals. In fact, a majority of recent arrivals—who were between the ages of 30 to 39, female, and currently married—were likely OWP holders married to Hong Kong natives. In terms of socioeconomic circumstances, the data showed lower status, e.g., lower education attainment, higher percent under poverty, and lower employment rate, among migrants, especially for those from Guangdong, but slightly better for recent arrivals. This could have been a reflection of the older demographic profile in the overall migrant group.

### 3.2. Comparison of Health Status

#### 3.2.1. Descriptive Statistics

Regardless of which outcome measure we used, migrants from Mainland China reported worse health status, in comparison with Hong Kong natives. The percent of migrants who self-reported fair/poor health status almost doubled what was reported among the natives, and migrants were also more likely to have been diagnosed with diabetes, hypertension, and coronary artery disease (Table 1). On the other hand, recent arrivals reported better health not only compared with migrant group as a whole, but better health than the natives, which seemed to support the “healthy migrant hypothesis”. 

Demographic differences between the comparison groups obviously affect the variation in health status. However, as shown in Table 2, when compared within each of the 10 age-sex category, migrants were still more likely to have reported “fair/poor” health status than the natives, with the exception of male migrants ages 30–39. We were not able to further test the “healthy migrant hypothesis” with age-sex specific analysis, due to the small sample size of recent arrivals.

**Table 1:** Demographics and socioeconomic conditions among Hong Kong residents and mainland migrants.

	Hong Kong	Migrants from Mainland China				
		All Migrants	By birth province		By length of stay	
			Guangdong	Other Mainland	<4 years	4 years or longer
Total number of persons	15,305	7,735	6,102	1,633	293	7,442
**Socio-demographics**						
Age 18–29 %	24.10% ***	11.00%	11.30% ***	10.00%	29.00% ***	10.30%
Age 30–39 %	19.60% ***	12.00%	8.50% ***	25.20%	42.00% ***	10.80%
Age 40–49 %	22.90% ***	16.50%	15.50% ***	20.20%	18.40% ***	16.40%
Age 50–59 %	19.50% ***	18.40%	19.70% ***	13.70%	6.5% ^ ***	18.90%
Age 60+ %	14.00% ***	42.10%	45.10% ***	30.90%	4.1% ^ ***	43.60%
Female %	49.90% ***	55.00%	53.40% ***	61.10%	73.40% ***	54.30%
Currently married %	55.10% ***	69.00%	68.10% **	72.30%	72.00%	68.90%
Education secondary and above %	28.70% ***	11.40%	8.60% ***	22.00%	18.40% ***	11.10%
Monthly personal income $4,000 and above %	66.00% ***	46.10%	44.30% ***	53.00%	44.40%	46.20%
Currently employed %	64.90% ***	41.00%	38.80% ***	49.20%	46.10% ***	40.80%
**Health Status**						
Self-reported fair/poor %	24.50% ***	40.50%	42.40% ***	33.70%	23.90% ***	41.20%
Current smoker %	13.50%	14.20%	14.60%	13.00%	12.30%	14.30%
Currently diagnosed with						
Diabetes %	3.30% ***	8.60%	9.20% **	6.60%	1.00% ***	8.90%
Hypertension %	8.80% ***	20.60%	22.20% ***	14.80%	2.70% ***	21.40%
Coronary artery disease %	0.40% ***	1.20%	1.30%	1.20%	0.30%	1.30%
Any of the above 3 chronic conditions %	10.50% ***	24.10%	25.80% ***	17.80%	3.40% ***	24.90%
**Utilization**						
Having usual source of care %	23.40% ***	17.80%	17.40%	19.20%	8.50% ***	18.20%
Average no. of doctor visits [Mean(SE)]	4.53 *** (0.05)	5.10 (0.09)	5.22 *** (0.10)	4.44 (0.16)	2.55 *** (0.29)	5.20 (0.09)
Average no. of visits to public sector [Mean(SE)]	0.99 *** (0.02)	2.18 (0.05)	2.27 *** (0.05)	1.84 (0.11)	0.79 *** (0.15)	2.23 (0.05)
Average no. of visits to private sector [Mean(SE)]	3.15 *** (0.04)	2.51 (0.06)	2.57 * (0.07)	2.30 (0.10)	1.46 *** (0.16)	2.55 (0.06)
Average no. of visits to A&E ^1^ [Mean(SE)]	0.04 *** (0.00)	0.09 (0.01)	0.09 (0.01)	0.37 (0.01)	0.04 ** (0.02)	0.09 (0.01)
Average ratio of visits to public sector ^2 ^[Mean(SE)]	0.19 *** (0.00)	0.42 (0.01)	0.44 (0.43)	0.36 (0.43)	0.26 *** (0.40)	0.43 (0.44)

Source: 2009 Thematic Household Survey, HK CS&D; Notes: ^ indicates that cell size is smaller than 30; * Differences between comparison groups are significant at *p* < 0.05, **p < 0.01, ***p<0.001; ^1^ Accident & Emergency Department; ^2^ Defined as the percent of total number of western medicine consultations that happened in the public sector.

**Table 2:** .Overall health status (% fair/poor) among Hong Kong residents and mainland migrants, by age and sex.

(Gender Age)		Hong Kong	Migrants from Mainland China											
			All Migrants	By birth province		By length of stay (year)								
				Guangdong	Other Mainland	<4	4 - 7	8–10	11–20	21–30	31–40	41–50	51–60	61+
Total		24.5% ***	40.5%	42.4% ***	33.7%	23.9% ***	26.3%	24.6%	31.2%	35.0%	45.1%	54.6%	58.4%	63.1%
Male	18–29	13.70% **	19.00%	18.00%	24.30% ^	28.00% ^	18.40%	12.50%	19.40%	25.50%				
	30–39	18.20%	16.60%	17.60%	15.20% ^	11.80% ^	22.20%	26.90%	20.90%	13.30%	7.10%			
	40–49	20.50% **	27.50%	27.40%	28.10%	29.20% ^	31.60%	22.50%	24.70%	26.80%	32.50%	37.50%		
	50–59	27.90% **	34.20%	34.30%	33.30%	25.00% ^	27.80%	27.30%	34.80%	30.70%	42.90%	31.00%	23.80%	
	60+	40.20% ***	57.40%	58.70% *	50.00%	50.00% ^	50.00%	57.90%	47.60%	52.20%	54.70%	57.50%	58.50%	65.00%
Female	18–29	17.40%	18.80%	17.50%	23.30% ^	20.00% ^	15.40%	15.60%	19.30%	28.10%				
	30–39	19.40%	21.60%	20.90%	22.40%	18.90% ^	24.90%	22.00%	23.00%	17.30%	17.60%			
	40–49	23.40% **	29.00%	30.10%	25.90%	30.00% ^	30.10%	23.40%	29.40%	28.60%	31.70%	66.70%		
	50–59	33.90% ***	41.70%	43.10%	35.40%	45.50% ^	40.90%	30.80%	43.90%	44.30%	39.80%	35.60%	41.90%	
	60+	46.70% ***	58.30%	59.20%	54.20%	50.00% ^	40.00%	52.20%	65.00%	51.50%	54.10%	59.70%	61.30%	61.20%

Source: 2009 Thematic Household Survey, HK CS&D; Notes: ^ indicates that cell size is smaller than 30; * Differences between comparison groups are significant at p < 0.05, ** p < 0.01, *** p < 0.001.

Looking at longer length of stay within each age-sex category, health generally deteriorated as the length of stay got longer. However, the data suggested a non-linear relationship between the two variables. As a whole, migrants from Guangdong reported worse health status, in comparison with migrants from other Mainland provinces, though most of the age-sex-specific differences were not statistically significant.

#### 3.2.2. Regression Results

Table 3 presented the logistic regression results for self-reported health status. We found that the age-sex adjusted odds of self-reporting fair/poor health were almost 1.5 times higher among Mainland migrants compared with Hong Kong natives. When additional covariates were included in the regression, the odds among Mainland migrants were slightly lower, but they were still 1.2 times more likely to report fair/poor health compared to the Hong Kong counterparts. As expected, the odds of reporting fair/poor health increased with age (OR = 4.47 for those ages 60 and above compared with those ages 18–29), decreased with household income, and were modestly higher among females (OR = 1.16) and current smokers (OR = 1.27), but lower among those married (OR = 0.81), with high-school or above education (OR = 0.88), and employed (OR = 0.79). 

**Table 3:** Results of the logit regression of self-reported poor health among Hong Kong adult residents.

	Age-Sex Adjusted Odds Ratio (95% Confidence Interval)	Full Model Odds Ratio (95% Confidence Interval)
Mainland Migrant	1.44 *** (1.35–1.53)	1.23 *** (1.10–1.39)
*(reference = Hong Kong Native)*		
Age *(reference=age 18–29)*		
30–39	1.19 ** (1.06–1.33)	1.37 *** (1.22–1.55)
40–49	1.55 *** (1.40–1.72)	1.77 *** (1.58–2.00)
50–59	2.45 *** (2.22–2.72)	2.71 *** (2.41–3.05)
60+	4.90 *** (4.44–5.41)	4.47 *** (3.98–5.01)
Female *(reference = male)*	1.19 *** (1.12–1.26)	1.16 *** (1.09–1.24)
Born in Guangdong province	--	1.14 * (1.01–1.28)
*(reference = other birthplaces in Mainland or Hong Kong natives)*		
Recent arrivals *(reference = Hong Kong natives or other migrants staying in Hong Kong for 4 years or longer)*	--	0.84 (0.63–1.11)
Currently married* (reference = not married)*	--	0.81 *** (0.75–0.87)
High school graduates	--	0.88 ** (0.81–0.96)
*(reference = Education below secondary)*		
Categorical monthly household income	--	0.96 *** (0.95–0.97)
Currently employed *(reference = unemployed)*	--	0.79 *** (0.73–0.85)
Current smoker *(reference = non-smoker)*	--	1.27 *** (1.16–1.39)

Source: 2009 Thematic Household Survey, HK CS&D; Note: -- indicates excluded variables; * Significant at *p* < 0.05, **p < 0.01, ***p < 0.001. N = 23,040.

Turning to the two migration factors, similar to earlier findings from the descriptive analysis, we found slightly higher odds of reporting fair/poor health among migrants from Guangdong in comparison to migrants from other Mainland provinces or Hong Kong natives (OR = 1.14). Although the recent arrivals had lower odds of reporting fair/poor health, supporting the hypothesis of “healthy migrant effect”, they were not statistically significant, perhaps due to the relative small number of recent arrivals included in the survey.

### 3.3. Comparison of Health Care Utilization

#### 3.3.1. Descriptive Statistics

In terms of utilization, migrants had more doctor visits compared with the natives, largely reflected in more visits to public clinics (Table 1). However, when looking within specific age-sex groups (Table 4), in comparison to the natives, both male and female migrant groups had lower average number of total visits at every age group except ages 60 and above, which showed the results from the migrant group as a whole having higher average number of doctor visits were entirely driven by the elderly population. In fact, we saw utilization among certain middle-age migrant groups (e.g., female ages 30–39, and male ages 40–49) fell much behind their native counterparts. Turning to the other comparison groups, Table 1 showed migrants from Guangdong having slightly higher average number of visits compared to other migrants. Age-sex-group-specific data in Table 4 confirmed this finding for most age groups, except males ages 18–49 and females ages 30–39; however, none of these differences is statistically significant. We also found that recent arrivals in most age-sex groups had fewer doctor visits compared to migrants with longer stays and to Hong Kong natives. However, similar to findings from Table 2, we did not see a clear linear increase in utilization with longer length of stay, and readers should interpret these results with caution due to the small sample size. 

Other utilization measures rendered similar results. Most notably, compared with Hong Kong natives, migrants from Mainland China, especially the recent arrivals, were less likely to have a usual source of care (Table 1). Migrants, as a whole group, were more likely to use A&E services, too.

#### 3.3.2. Regression Results

The negative binomial regression results confirmed most of the above findings concerning difference in total number of doctor visits between Mainland migrants and Hong Kong natives (Table 5). After adjusting for age, sex, and other socioeconomic and health characteristics, we still found migrants had significantly fewer doctor visits than the natives (Coefficient = −0.21). All but two coefficients (for marital and employment status) were statistically significant, pointing to the direction as we anticipated—older, less healthy individuals with higher incomes tended to visit doctors more frequently. Most notably, controlling for the other factors, migrants from Guangdong province who presumably spoke the same language as the Hong Kong natives, had more visits (Coefficient = 0.10); whereas recent arrivals who stayed in Hong Kong for three years or less had fewer (Coefficient = −0.42). This seemed to suggest that language familiarity was a facilitator for the migrants to integrate into the new system and gain better access to health care. On the other hand, it might take a long time for the new migrants to do so. 

**Table 4:** Average number of doctor visits in the last 12 months among Hong Kong residents and mainland migrants, by age and sex.

	Hong Kong	Migrants From Mainland China											
		All Migrants	By Birth Province		By Length of Stay								
			Guangdong	Other Mainland	<4 years	4–7	8–10	11–20	21–30	31–40	41–50	51–60	61+
Male													
18–29	2.66 *	1.79	1.74	2.07	1.72 ^*	0.92	1.67	1.78	3.04				
30–39	3.48	2.85	2.76	2.97	0.88 ^	1.44 ^	2.46 ^	2.91	3.42	2.68 ^			
40–49	4.06 **	2.80	2.72	3.04	1.83 ^**	3.53 ^	1.23	2.53	3.22	2.61	7.38 ^		
50–59	4.18	3.45	3.57	2.66	2.63 ^	2.61 ^	1.73 ^	4.04	2.84	3.75	5.07	3.00 ^	
60+	5.56 **	6.51	6.61	5.96	0.75 ^*	7.13 ^	9.16 ^	4.17	5.43	6.32	6.24	7.30	6.74
Female													
18–29	3.68	3.06	3.17	2.67	1.95	2.38	2.73	3.60	4.16				
30–39	5.34 **	3.78	3.77	3.80	2.63 ***	3.06	3.27	4.15	5.67	6.88 ^			
40–49	5.28 ***	3.97	4.07	3.67	2.93 **	2.63	4.64	3.70	4.16	6.10	4.42 ^		
50–59	5.87	5.67	5.69	5.54	4.27 ^	4.05 ^	2.94	5.29	6.38	6.43	6.19	6.00	
60+	6.78 **	7.68	7.81	7.01	11.25 ^*	3.13 ^	6.11	5.71	6.76	7.48	7.78	8.71	8.22

Source: 2009 Thematic Household Survey, HK CS&D; Notes: ^ indicates that cell size is smaller than 30; * Differences between comparison groups are significant at p < 0.05, ** p < 0.01, *** p < 0.001.

**Table 5:** Regression results of health care utilization among Hong Kong adult residents.

		Total number of doctor visits	Percent of visits to western medicine doc in the public sector	Having usual source of care
		Coefficient (95% Confidence Interval)	Coefficient (95% Confidence Interval)	Odds Ratio (95% Confidence Interval)
Mainland Migrants *(reference = Hong Kong Native)*		−0.21 *** (−0.27, −0.15)	0.54 *** (0.42, 0.66)	0.78 *** (0.69, 0.90)
Age *(reference = age 18–29)*				
	30–39	0.31 *** (0.26, 0.36)	0.12 (-0.03, 0.27)	1.37 *** (1.22, 1.54)
	40–49	0.33 *** (0.28, 0.39)	0.53 *** (0.40, 0.67)	1.57 *** (1.39, 1.77)
	50–59	0.41 *** (0.36, 0.47)	1.05 *** (0.92, 1.19)	1.36 *** (1.20, 1.54)
	60+	0.72 *** (0.67, 0.78)	1.36 *** (1.22, 1.49)	1.55 *** (1.36, 1.76)
Female *(reference = male)*		0.26 *** (0.23, 0.29)	−0.20 *** (−0.27, −0.13)	1.21 *** (1.13, 1.30)
Born in Guangdong province *(ref = other birthplaces in Mainland or Hong Kong natives)*		0.10 ** (0.04, 0.16)	−0.11 (−0.24, 0.01)	0.95 (0.83, 1.10)
Recent arrivals *(reference = Hong Kong natives or other migrants staying in Hong Kong for 4 years or longer)*		−0.42 *** (−0.56, −0.28)	0.12 (−0.23, 0.48)	0.46 *** (0.30, 0.71)
Currently married * (reference = not married)*		0.03 (0, 0.06)	−0.12 ** (−0.19, −0.04)	1.16 *** (1.07, 1.26)
High school graduates *(reference = Education below secondary)*		0.10 *** (0.06, 0.14)	−0.32 *** (−0.42, −0.22)	1.52 *** (1.40, 1.65)
Categorical monthly household income		0.01 *** (0.01, 0.02)	−0.11 *** (−0.12, −0.09)	1.11 *** (1.09, 1.12)
Currently employed *(reference = unemployed)*		0.03 (0, 0.07)	−0.58 *** (−0.67, −0.50)	1.02 (0.94, 1.11)
Current smoker *(reference = non-smoker)*		−0.14 *** (−0.18, −0.10)	−0.12 * (−0.22, -0.12)	0.87 ** (0.78, 0.96)
Self-reported poor health * (reference = good or excellent health)*		0.55 ***(0.52, 0.59)	0.60 *** (0.53, 0.67)	1.82 *** (1.70, 1.96)
Total number of visits to western medical doctors		--	0.003 (−0.001, 0.008)	--

Source: 2009 Thematic Household Survey, HK CS&D; Note: -- indicates excluded variables; * Significant at *p* < 0.05, ** *p* < 0.01, ***p < 0.001. N = 23,040; Total number of doctor visits were estimated using negative binomial regression, percent of visits to western medicine doc in the public sector were estimated using generalized linear model, and having usual source of care were estimated using logit model.

When looking into the choice of public and private doctors for those who had at least one visit to the western medicine clinics, we found that Mainland migrants were more likely to choose public doctors (Coefficient = 0.54), so were elders and those reported poor health. Females and those who were married with higher income were less likely to choose public doctors. These differences largely reflect the public-private split of the current health care system in Hong Kong, where everyone has access to the almost-free public health services and only those who could afford would use more private services. Mainland migrants, like other vulnerable populations in Hong Kong, depend on the public sector as a safety net. Controlling for other covariates, we did not find any significant results for migrants from Guangdong or recent arrivals. 

In addition to the actual utilization of services, we also examined if there is any self-reported difference in having a usual source of care—a defining feature of strong primary care—between migrants and natives. Again, we found significantly lower odds of having a usual source of care among Mainland migrants in comparison to Hong Kong natives (OR = 0.78). The odds were especially low for recent arrivals (OR = 0.46), suggesting they were as half likely to have usual source of care as others. We did not see a linear relationship between age and usual source of care, but those with poor health were more likely to report having usual source of care, so were female and those more socially advantaged. 

## 4. Discussion and Conclusions

Migration is an important social determinant of health. Despite a growing field of literature on international migrants’ health, few studies have looked at health and health care among internal migrants. When they did, most have focused on migrants moving from rural to urban, as a result of a country’s economic development. Rarely was there an opportunity to look at a situation falling between international and purely internal migration—individuals with the same ethnicity migrating across a much more significant administrative border through a much more stringent process than moving from one city to another within a country. Using data from Hong Kong, this study provides a comprehensive analysis of health and health care utilization among migrants from Mainland China in comparison with natives in Hong Kong. It is, to our knowledge, the first such study in Hong Kong. It may also shed light on understanding similar migration phenomena in other countries or regions, such as eastern European countries, where migrants with similar background have to face very different health care system in the destination. 

In summary, we found migration to be a significant predictor of health and health care among residents in Hong Kong. Overall, Mainland migrants reported worse health condition and fewer doctor visits than Hong Kong natives, after controlling for other socio-demographic factors. Migrants were also less likely to have a usual source of care and relied more on the public sector in Hong Kong’s mixed health care system. Within the migrant group, migrants from Guangdong province who presumably spoke the same language as the Hong Kong natives, had significantly more doctor visits, suggesting language familiarity might be an important facilitator that helped migrants to better understand the health care system and to more easily communicate with health service providers. On the contrary, recent arrivals who stayed in Hong Kong for three years or less had fewer visits and were less likely to have a usual source of care, signaling they were the group that probably needed the most help to settle in the new environment. These findings, together with our analysis by age and sex highlight the importance of recognizing the different characteristics and therefore, needs, among subgroups within the migrant population. 

In terms of policy implications, our study suggests that migrants from Mainland China, especially the recent arrivals, represent an under-privileged vulnerable population in Hong Kong. It requires a combination of targeted social and health policies to help them integrate into society, improve socioeconomic status, understand the complex health care system, and thus advance their overall health and access to care. In the current primary care reform, more resource and attention need to be devoted to migrants, especially to the subgroups that were found to lag far behind their native counterparts in service utilization, for example, those who are middle-aged and those who do not speak Cantonese. Recognizing the heterogeneity of the migrant group, programs such as health education campaign or subsidized service delivery should also be tailored to serve varying needs from different migrant groups (e.g., recent *vs.* earlier arrivals, younger *vs.* older, highly- *vs.* lowly-educated, *etc*). 

We only found weak evidence supporting the “healthy migrant hypothesis”—recent arrivals were less likely to report poor health, and the odds of reporting poor health increased with length of stay, but due to the small sample size of recent arrivals in the survey, we did not find the difference to be statistically significant. Other researchers have questioned the healthy migrant hypothesis based on reasons such as a cohort effect in addition to length of stay effects [32], recall bias of survey respondents [1], and the health status of recent arrivals should be compared to that of people from place of origin and not natives [33]. Some of these explanations may also apply here. Most notably, we did have a relatively small sample size for recent arrivals (n = 293), and once divided into 10 age-sex categories, most of the categories ended up having fewer than 30 observations, too few for us to draw any conclusion on. The inherent limitation of a cross-sectional survey also hampered our ability to draw more definitive conclusion regarding the healthy migrant hypothesis [11]. 

There are several other caveats worth mentioning. As in any household survey, most of the variables, particularly, measures of health care utilization were self-reported, and could suffer from recall bias. To capture the length of stay, the survey only asked the question of “for how many *years* have you been living in Hong Kong”. Therefore, we didn’t have more detailed information to identify the “newest” arrivals who might have only migrated for less than a year, neither could we distinguish people who stayed for 13 months from people who stayed for 23 months (both of whom could have responded one year). We also lacked a direct measure of language familiarity, since the question was not asked in the survey. We used being born in Guangdong province as a proxy for language, which may not be appropriate as respondents could have moved to another province at an early age and thus do not speak Cantonese.
